# Supplementary figures and images for: Novel real-time PCR based assays for differentiating fall armyworm strains using four single nucleotide polymorphisms
Source: PeerJ. 2021 Sep 24;9:e12195. doi: 10.7717/peerj.12195 (PMC8475543; doi:10.7717/peerj.12195)

# SNP A

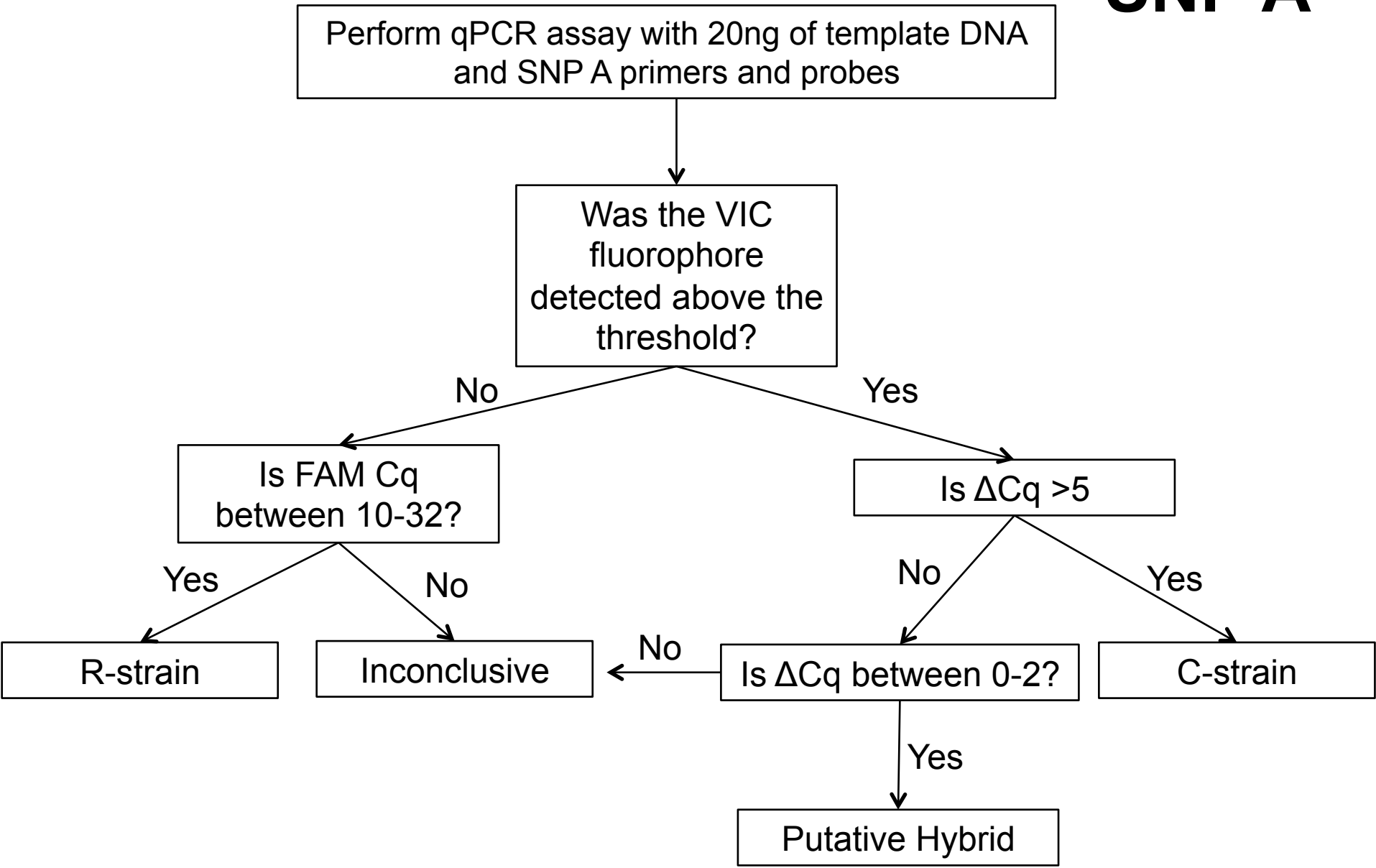

Supplement: Supplemental Information 9 [file peerj-09-12195-s009.pdf]

# SNP B

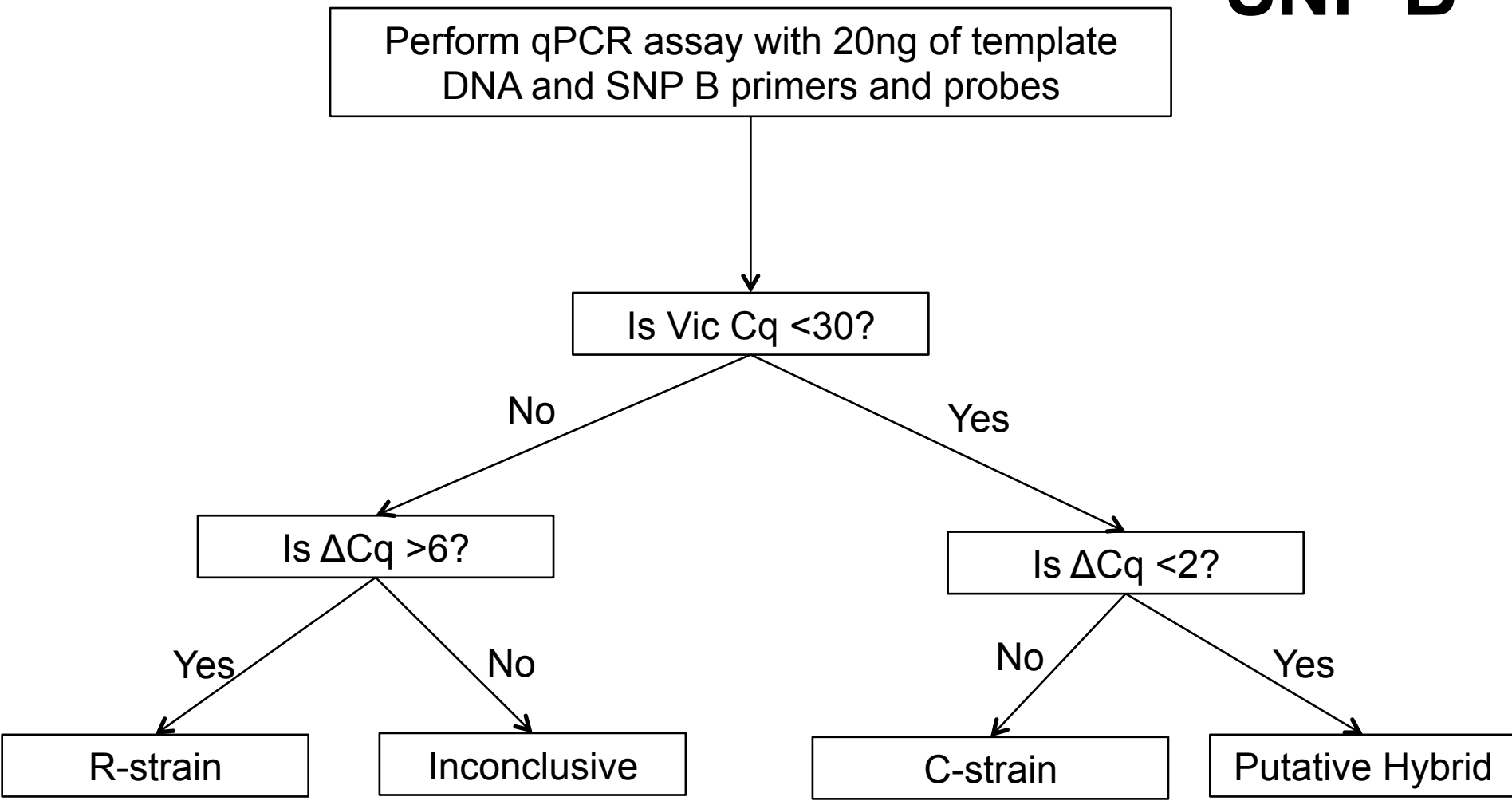

Supplement: Supplemental Information 10 [file peerj-09-12195-s010.pdf]

# SNP C

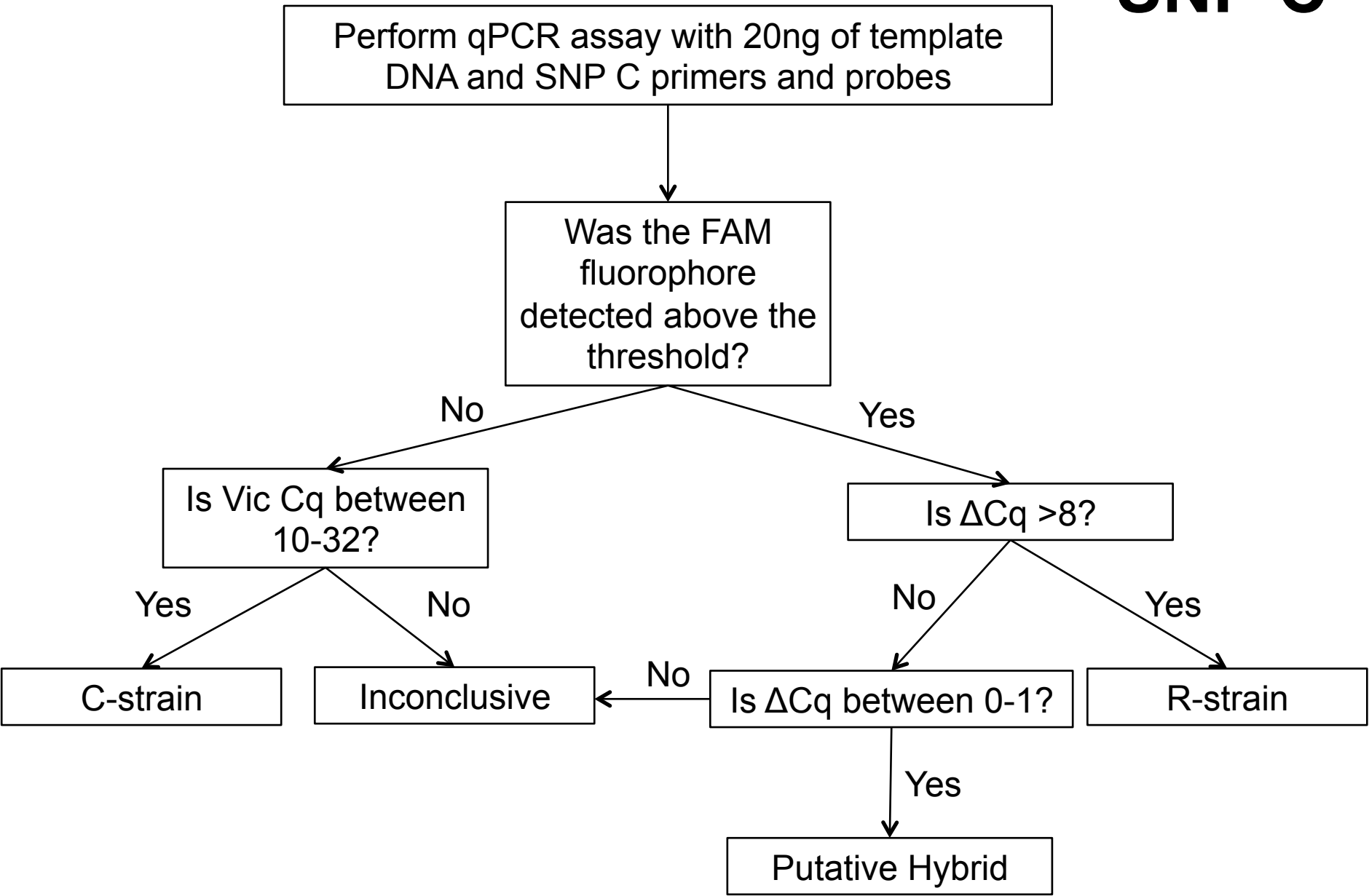

Supplement: Supplemental Information 11 [file peerj-09-12195-s011.pdf]

# SNP D

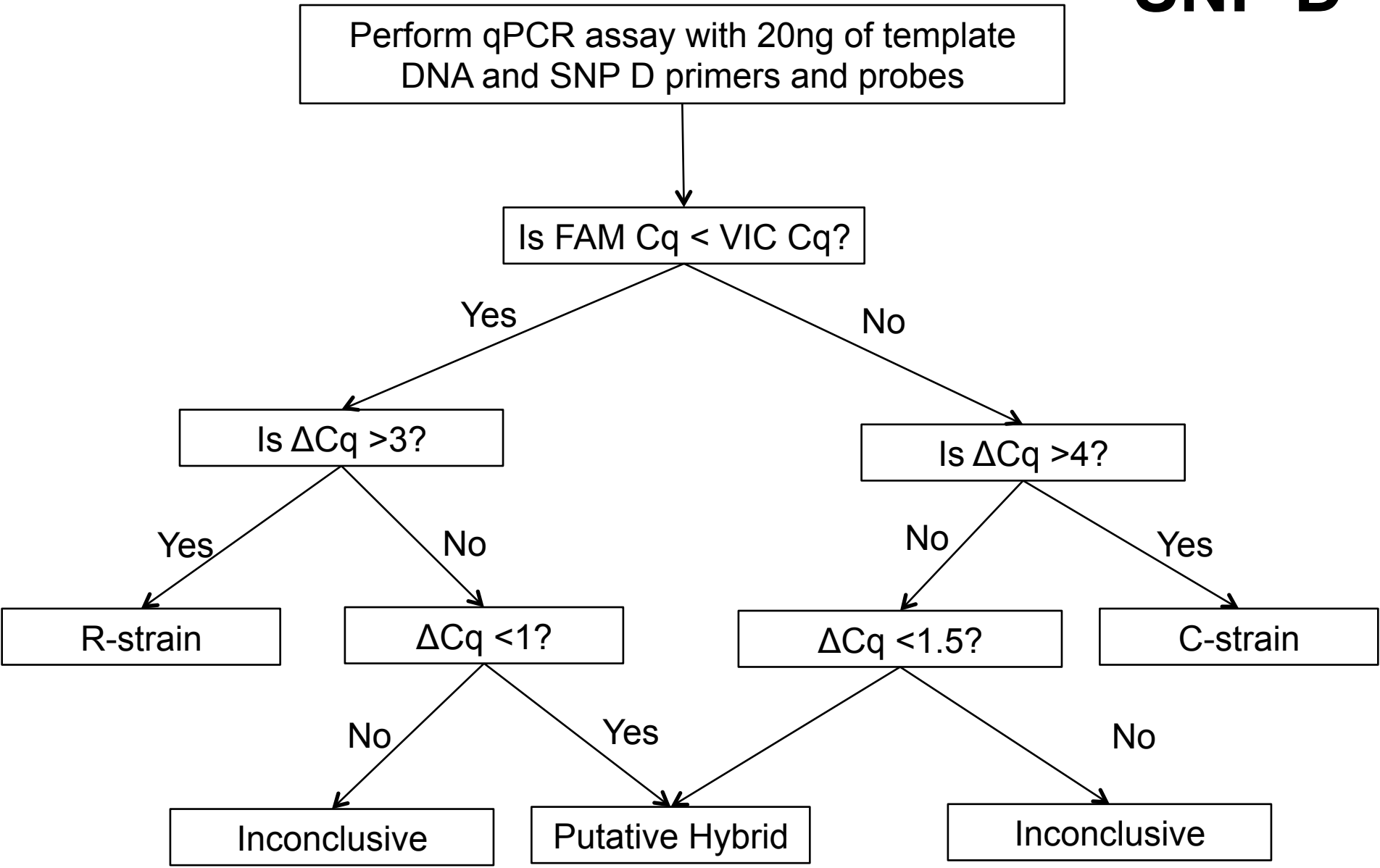

Supplement: Supplemental Information 12 [file peerj-09-12195-s012.pdf]

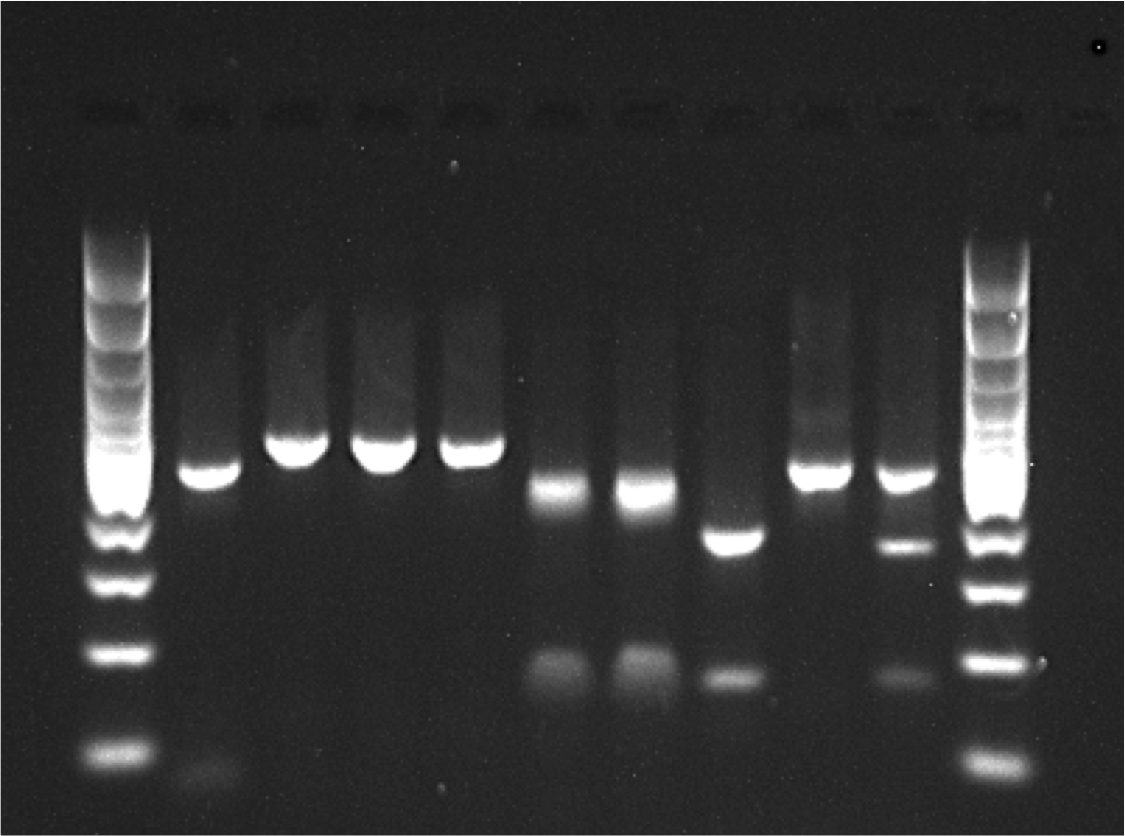

Supplement: Supplemental Information 13 [file peerj-09-12195-s013.png]
